# Method of determining geometric patient size surrogates using localizer images in CT

**DOI:** 10.1002/acm2.12814

**Published:** 2020-01-28

**Authors:** Christiane S. Burton

**Affiliations:** ^1^ Boston Children's Hospital Boston MA USA; ^2^ Harvard Medical School Boston MA USA

**Keywords:** AAPM 204, AAPM 220, computed tomography, CTDIvol, dose, effective diameter, SSDE

## Abstract

**Purpose:**

Size‐specific dose estimates (SSDE) requires accurate estimates of patient size surrogates. AAPM Report 204 shows that the SSDE is the product of CTDIvol and a scaling factor, the normalized dose coefficient (NDC) which depends on patient size surrogates for CT axial images. However, SSDE can be determined from CT localizer prior to CT scanning. AAPM Report 220 charges that a magnification correction is needed for geometric patient size‐surrogates. In this study, we demonstrate a novel “model‐based” magnification correction on patient data.

**Methods:**

573 patient scans obtained from a clinical CT system including 229 adult abdomen, 284 adult chest, 48 pediatric abdomen, and 12 pediatric chest exams. LAT and AP dimensions were extracted from CT localizers using a threshold extraction method (the ACR DIR). The model‐based magnification correction was applied to the AP and LAT dimensions extracted using the ACR DIR. NDC was calculated using the effective diameter for the ACR DIR only, the model‐based localizer‐based and axial‐based approaches. The LAT and AP dimensions were extracted from the “gold” standard CT axial scans. Outliers are defined as points outside the 95% confidence intervals and were analyzed.

**Results:**

NDC estimates for the localizer‐based model‐based approach had an excellent correlation (R^2^ = 0.92) with the gold standard approach. The effective diameter for ACR DIR and model‐based approaches are 8.0% and 1.0% greater than the gold standard respectively. Outliers were determined to be primarily patient truncation, with arms down or with devices. ACR DIR size extraction method fails for bariatric patients where the threshold is too high and some of their anatomy was included in the CT couch, and small patients due to the CT couch being included in the size measurement.

**Conclusion:**

The model‐based magnification method gives an accurate estimate of patient size surrogates extracted from CT localizers that are needed for calculating NDC to achieve accurate SSDE.

## 1 Introduction

Dose from medical use has increased from ~15%‐50% from the 1980s to 2006 with CT now representing 50% of this dose.^1^ Keeping within the As Low As Reasonably Achievable (ALARA) principles is still a challenge for clinical staff including radiologists and medical physicists.^2^ Quantifying absorbed dose to the patient is necessary. The CTDIvol only represents the radiation output of a system for specific sets of conditions.^3^, ^4^, ^5^, ^6^, ^7^, ^8^ A method that scales CTDIvol with a scaling factor that depends on patient size exists. The American Association of Physicists in Medicine (AAPM) Report 204^8^ introduced this scaling factor, the normalized dose coefficient (NDC), and it is calculated based on patient size surrogates anterior‐posterior (AP), lateral (LAT), and effective diameter (sqrt[AP*LAT]). However, these estimates of patient surrogates from CT axial images can only be performed after the CT scan is finished and images are reconstructed. It would be useful to have knowledge of patient surrogates and size‐specific dose estimates (SSDE) prior to scanning, and this can be achieved using CT localizer images. AAPM 220 charges that four sources of error be taken into account when extracting attenuation‐based size surrogates from the CT localizer. However, three of four of these sources only need to be taken into account for patient size surrogate WED because it depends on patient attenuation. For this study we focus solely on the geometric size surrogates and therefore only require a magnification correction to the AP and LAT dimensions.

**Figure 1:**
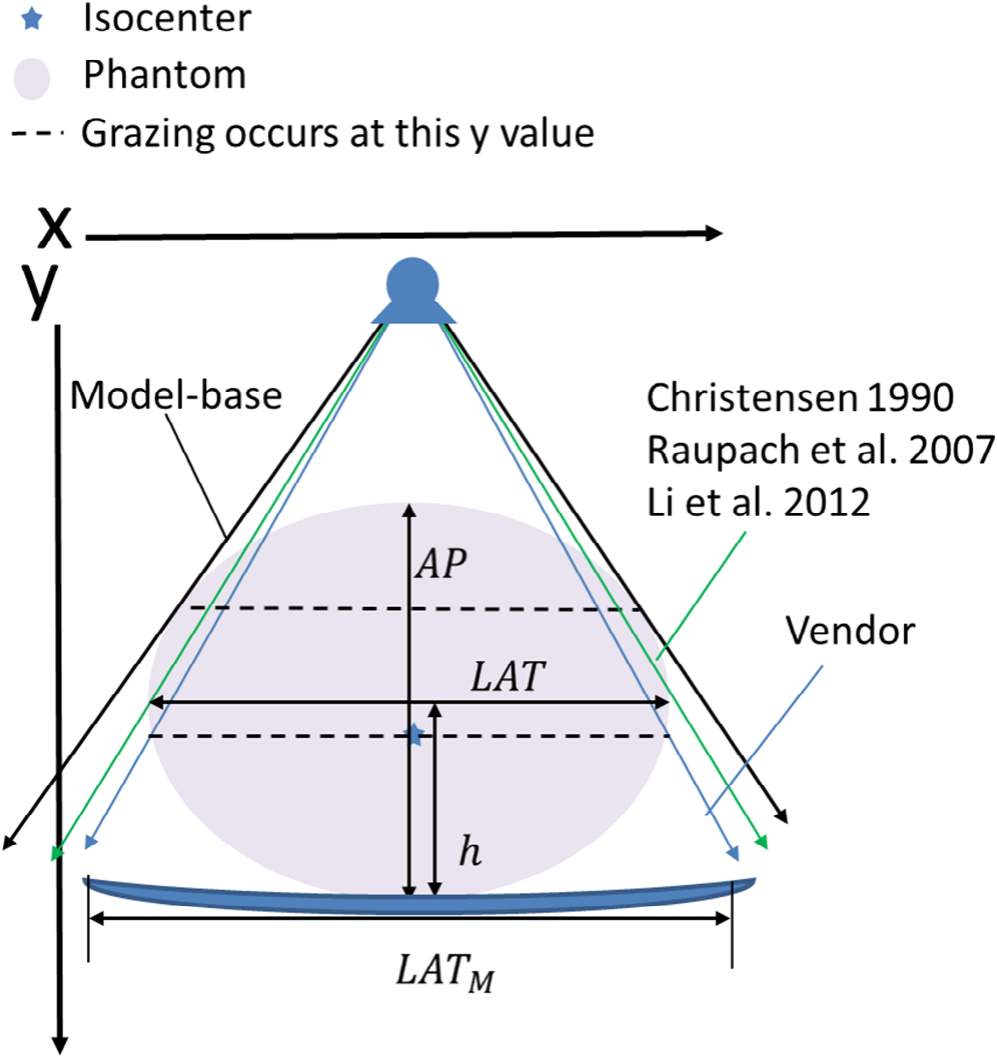
Image showing how the x‐rays graze the anatomy at a point higher than the greatest lateral width.

In a previous study conducted in our laboratory, we demonstrated a magnification/minification approach that takes into account how the edges of the anatomy are actually projected onto the image plane for both the LAT and AP dimensions.^9^ These assumptions were different from other known methods 10, 11, 12, 13, 14 and the typical vendor's method which all use similar triangles to calculate the LAT and AP dimensions. The vendor's method performs a SID/SOD correction. The previous methods extend the vendor's approach by including a table offset where they assume that the x‐ray intersecting the patient is at their widest extent, which is incorrect (Table 1). The model‐based magnification method approach assumes that the patient is an ellipse, and the first point of intersection between the patient and x‐ray is taken into account.^9^ This is because the patient's widest points as shown on the image are actually in‐line with the x‐ray projected at a point of contact on the patient that is not necessarily the widest point, as shown in Fig. 1. The approach was validated using elliptical phantoms placed at different table heights while centered in the x‐direction. Table II of Burton et al.^9^ demonstrated that the model‐based method provides consistent accurate results, less than 1.8% of maximum error for absolute size for all measurement conditions relative to 30.9% and 7.5% for the vendor and Christensen/Raupach/Li approaches respectively. Using the model‐based magnification correction approach, the patient size surrogates yield the best estimate of the actual dimension.

In this article, we evaluate our model‐base magnification/minification correction of AP and LAT for NDC calculations on patient data.

## 2 Method

### 2.1 Data collection

The following data were collected under a protocol that was IRB approved retrospectively. There were 573 patients included in this analysis. The patient data were collected from four different CT axial and localizer data sets are: (1) 229 routine adult abdomen/ pelvis scans, (2) 284 adult chest scans, (3) 48 pediatric abdomen/pelvis scans, and (4) 12 pediatric chest scans. For all CT exams, the kernel which uses “STANDARD'' (vendor specific name that refers to a soft tissue reconstruction kernel), the Reconstruction Option was set to PLUS, and the ASiR Level is 40%. These data were acquired from a 64‐slice CT scanner from the same manufacturer (Optima CT660 scanner models from GE HealthCare, Chicago IL). Since this study looks at geometric patient size surrogates, the technique used in all cases is irrelevant. The following are parameters used for these scans.

**Table 1:** Experimental data collection of human patients of routine adult abdomen, adult chest, pediatric abdomen, and pediatric chest.

Data set	kV	NI	Pitch	Slice thickness (mm)
Adult abdomen/pelvis	120	15	0.52	5.0
Adult chest	120	15	1.38	5.0
Pediatric abdomen/pelvis	80	12	0.52	5.0
Pediatric chest	100	12	0.52	5.0

The parameters displayed are the kilovoltage peak (kV), the Noise Index (NI) which refers to a vendor specific automatic exposure control setting, the pitch (table distance traveled in one 360 gantry rotation divided by beam collimation), the slice thickness (mm), the slice interval. Not shown is the kernel which uses “STANDARD'' (vendor specific name that refers to a soft tissue reconstruction kernel), the Reconstruction Option was set to PLUS, and the ASiR Level is 40% for all of the data shown in the table.

### 2.2 The American College of Radiology dose index registry for data extraction from CT localizers

The American College of Radiology Dose Index Registry (ACR DIR) method allows facilities to compare their SSDE to both regional and national values based on patient size. An adaptive threshold algorithm is recommended for extracting patient dimensions AP_M_ and LAT_M_ (M denotes measured) which is defined here as the anterior‐posterior and lateral dimensions measured from the CT localizer images. The ACR DIR method has been applied and validated in a previous study^15^ for the purpose of extracting LAT_M_ and AP_M_ from patient data. For both AP and LAT CT localizer projections, for each z‐location within the CT axial scan length, a line profile is generated for each row in the CT localizer. A Savitzky‐Golay smoothing edge‐preserving filter is applied to the line profile for each row in order to smooth out small abrupt changes in pixel values without blurring the edges on the side of the patient. The upper and lower 5% of the pixel values are saturated to reduce the effect of image noise. A threshold of 30% of the maximum pixel value is set to exclude the thickness of the table for most patients. The algorithm counts the number of pixels that exceed the threshold to determine the patient's AP_M_ or LAT_M_ thickness at that particular z‐location. This process is repeated for all z‐locations over the same scan range of the axial CT scan.

### 2.3 Magnification correction methods

The model‐based method Burton et al.^9^ derived a magnification method for calculating the LAT and AP dimensions of the patient where the equations are reduced in final form (shown in their Appendix). These equations were derived for both LAT and AP using LAT_M_ and AP_M_ from the ACR DIR extraction approach as input with table height, h, and source‐to‐object distance (SOD) which can both be obtained from the DICOM header. The LAT and AP dimensions were extracted from CT images using a threshold method developed by Burton et al.^9^. The CT image will give the true dimension of the patient which is why it will be used here as the “gold” standard for comparing LAT and AP dimensions, and NDC calculations which use these dimensions.

The LAT and AP were plotted for all patient data with and without the model‐based approach as a function of the “gold” standard CT axial approach. We took the average thickness of the patient for ACR DIR, model‐based and CT scans can then be determined over the area of interest. Ideally, the measurements obtained using the model‐based method should be 1:1 with the CT axial‐based measurements; therefore we have added a line of unity which represents the ideal case for comparison was added to each plot. The AAPM report 204 showed D_E_ as function of AP, LAT, and LAT + AP for the purpose of estimating one parameter from another for the purpose of estimating the patient dose using a Monte Carlo (MC) or MC‐derived patient dose calculation. These measurements were taken at the University of Wisconsin‐Madison, so we labeled our results as “UW Madison”. We compared the “UW Madison” results to the AAPM report 204 by plotting the lines of best fit for our results of D_E_, calculated as √LAT⋅AP as a function of (AP + LAT)/2, LAT and AP, and overlapping the lines of best fit to the ones in the AAPM Report 204. Last, we calculate the NDC using D_E_ estimates from CT localizer images and CT axial scans and plotted localizer‐based as function of axial‐based NDC.

### 2.4 Data analysis

To analyze the data, a linear fit command (*polyfit* function from MATLAB, The Mathworks INC, Natick MA) was applied a first order linear fit and 95% confidence intervals with a linear regression (R^2^) for all data points combined. A tight 95% confidence interval means that the data will show that many of the data points will be clustered around the mean. The confidence interval is reported in millimeters for the x‐direction and unitless for the y‐direction and this number is the distance from the trendline to the confidence interval. Points that lie outside of this confidence interval are defined as outliers and examples of these cases are analyzed to characterize deviations from the correlations show in the AAPM TG reports. All data are included within this analysis and different data sets are labeled with different markers.

## 3 Results

### 3.1 LAT and AP comparison

**Figure 2:**
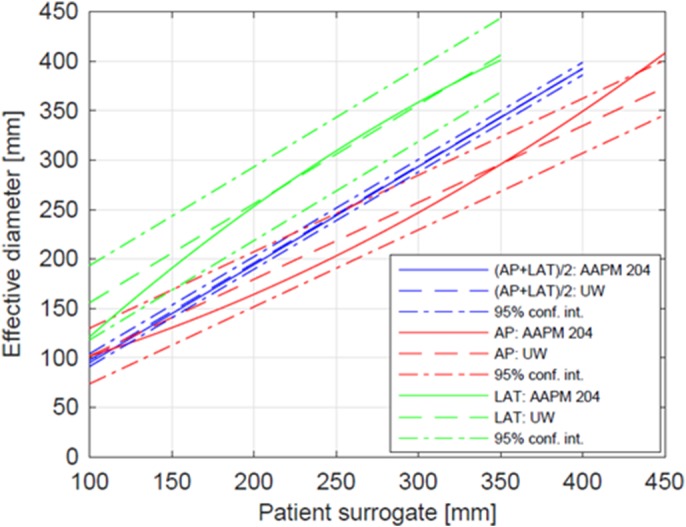
Localizer‐based dimension [mm] as a function of gold standard CT axial dimensions for (a) lateral and (b) anterior‐posterior dimensions using the ACR DIR method and model‐based method. The ACR DIR thresholding‐based size method fails for (a) bariatric patients due the patient information being below the threshold at the side thereby artificially decreasing the LAT dimension and (b) pediatric patients for the table being included with the patient dimension thereby artificially increasing the AP dimension.

Figure 2(a) shows LAT from model‐based magnification correction and LAT_M_ from the ACR DIR method as a function of CT axial LAT measurement. The linear regression demonstrates good correlation of LAT as a function of axial CT LAT (R^2^ = 0.86, 95% confidence interval range of ~51 mm) meaning that applying the model‐based magnification method will give an excellent estimate of the patient's LAT provided the ACR DIR method is given a patient that is not bariatric. Figure 2(b) shows AP from the model‐based magnification correction and AP_M_ from the ACR DIR as a function of “gold” standard CT axial AP measurement. The linear regression demonstrates excellent correlation of AP as a function of axial CT AP (R^2^ = 0.90, 95% confidence interval range of ~49 mm) meaning that AP will generally give an excellent estimate of the patient's AP provided that the ACR DIR method thresholds away the couch.

### 3.2 AAPM Report comparisons

**Figure 3:**
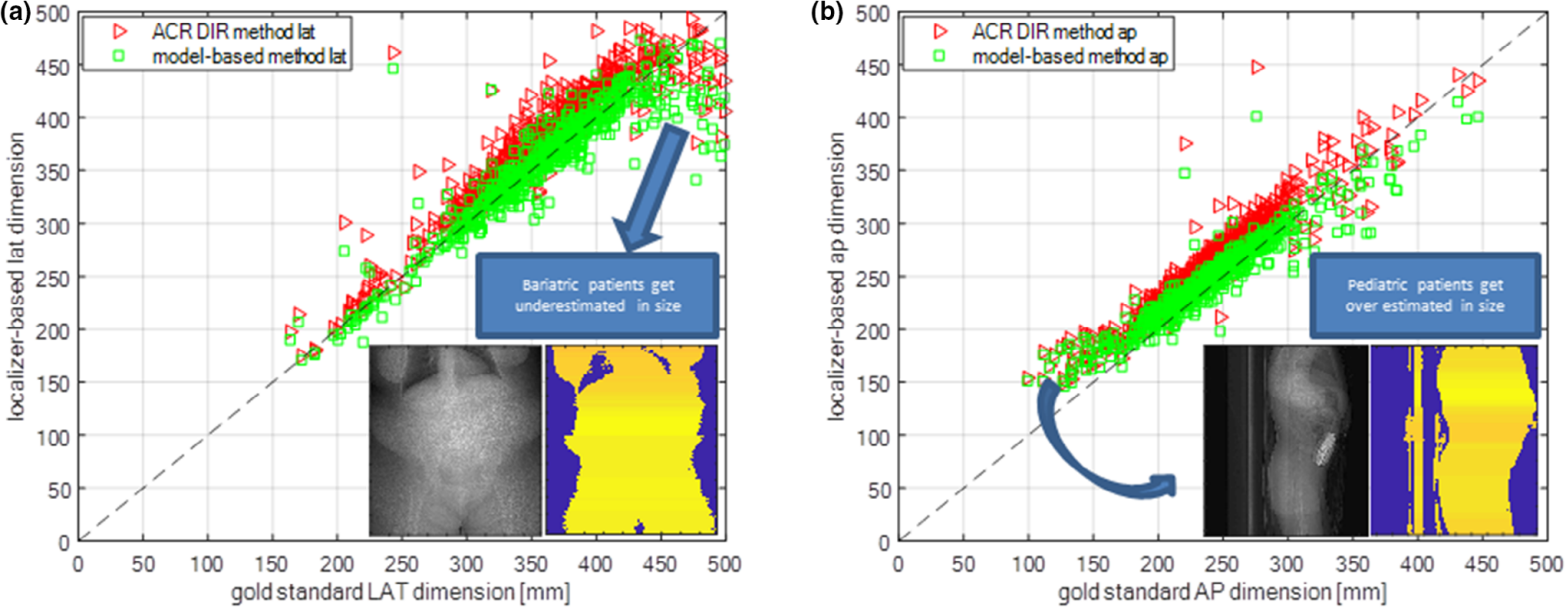
Comparing the fits from AAPM Report 204 for DE as a function of patient size surrogates (AP + LAT)/2 (blue lines), AP (green lines), and LAT (magenta lines), University of Wisconsin‐Madison (UW) first‐order fit (dotted‐dashed lines) and 95% confidence interval (colored dashed lines).

Figure 3 shows both the University of Wisconsin‐Madison (UW) fit and AAPM Report 204 fits of DE as a function of AP, LAT, and (AP + LAT)/2. It is shown that the AAPM fit falls within the 95% confidence interval.

### 3.3 NDC comparisons

**Figure 4:**
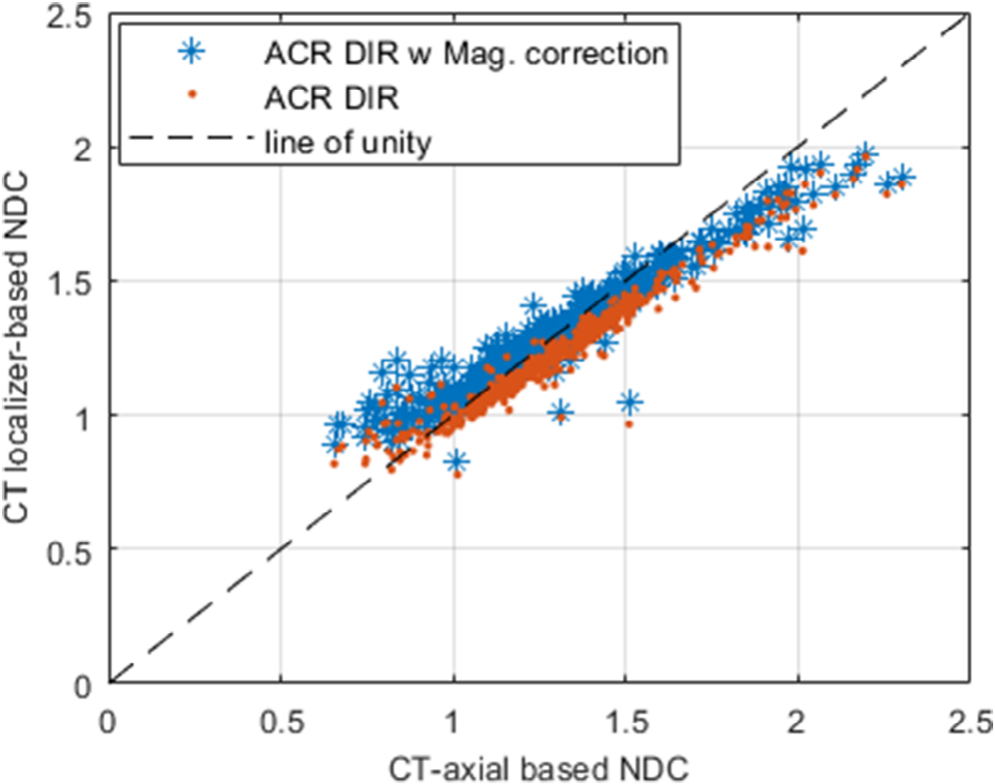
Localizer‐based normalized dose coefficient as a function of gold standard normalized dose coefficient (NDC) for model‐based (*blue astrices*) and ACR DIR (*red dots*) approaches.

Figure 4 shows localizer‐based NDC calculations for both model‐based and ACR DIR extraction only methods as a function of CT axial “gold” standard NDC. The linear regression demonstrates excellent correlation of NDC as a function of axial CT NDC (R^2^ = 0.91, 95% confidence interval range of ~ 2.082 × 10^−6^) meaning that, with exception of bariatric and thin pediatric patients, the model‐based magnification correction NDC will generally give an excellent estimate of CT axial gold standard NDC. Table 2 shows the linear regressions and R^2^ for NDC, LAT and AP measurements for magnification method and Duke only method as a function of axial‐based measurements.

**Table 2:** The linear fits and R^2^ for the normalized dose coefficients (NDC), lateral (LAT), and anterior‐posterior (AP) measurements for both the magnification method and Duke only method as a function of axial‐based measurements.

	Linear Fit	R^2^
NDC	*y* = 0.67902*x* + 0.38955	0.94
NDC ACR DIR	*y* = 0.69583*x* + 0.27993	0.9
LAT	*y* = 0.81464*x* + 68.4109	0.71
LAT ACR DIR	*y* = 0.88578*x* + 57.2124	0.66
AP	*y* = 0.62174*x* + 94.8361	0.7
AP ACR DIR	*y* = 0.648*x* + 136.2529	0.64

## 4 Discussion

For a more personalized approach in determining patient dose from CT, the model‐based magnification correction of LAT_M_ and AP_M_ dimensions from CT localizers to LAT and AP shows that NDC may be accurately calculated provided that LAT_M_ and AP_M_ extracted using the ACR DIR method is accurate. Using patient surrogates from CT localizers is beneficial because they may be included into data‐driven clinical work flows such as size adaptive protocol selection like diagnostic reference ranges (DRRs) which provide a minimum estimated patient dose. Additional benefits include reduced data overhead if CT axial images are not stored and errors related to axial calculation. Estimating geometric patient surrogates from CT localizers would require a magnification method.^16^

For all clinical data shown in Fig. 3, using the model‐based magnification correction shows excellent agreement with the CT axial “gold” standard for lateral and AP dimensions. In Fig. 3(a), on average the LAT_M_ for ACR DIR and LAT for model‐based are 6.0% greater and 0.14% less than the “gold” standard respectively. In Fig. 3(b), on average AP_M_ for ACR DIR and AP for the model‐based method are 11.0% and 2.0% greater than the gold standard respectively. Figure 3 shows that the UW linear fits of D_E_ as a function of (AP + LAT)/2, LAT, and AP compare well to the fits in the AAPM Report 204.^8^ The phantoms used by Boone et al. and Strauss et al. had circular cross‐sections whereas the Monte Carlo Voxelized Phantoms used by ICRU92 were elliptical. The LAT dimensions over 400 mm from the AAPM Report 204 are the only data outside of our 95% confidence interval. These data agree well with Burton and Szczykutowicz.^17^ which demonstrate a similar result using CT axial scans. In Fig. 4 there is excellent correlation of model‐based magnification correction with the “gold” standard NDC. On average, the NDC for ACR DIR and model‐based method are 10% greater and 0.8% greater than the “gold” standard respectively.

We explored the outlier points that fell outside of the 95% confidence intervals in Figs. 2(a) and 2(b). The most prevalent outliers are those where the ACR DIR either underestimates Fig. 3(a) or overestimates Fig. 3(b) the patient size for bariatric and pediatric cases, respectively. Figure 3(a) shows the bariatric patients with LAT dimensions roughly between 450‐500 mm do not continue the linear trend and fall outside of the 95% confidence interval. This is because the ACR DIR method failed for bariatric patients due to underestimation that some of their anatomy was classified as belonging to the CT couch and thresholded away. Figure 3(b) shows that pediatric patients with AP dimensions roughly between 100‐150 mm do not continue the linear trend and tend to fall outside of the 95% confidence interval. This is because the ACR DIR method fails for small patients due to the CT couch being included in the size measurement.

**Figure 5:**
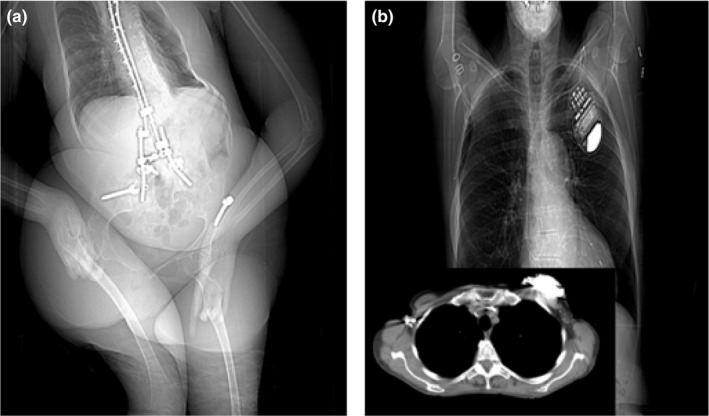
Other examples of how ACR DIR threshold algorithm causes an outlier case. Shown here are CT localizers where (a) the arms down and (b) a device is resting on a patient that will only be included in the CT axial scan extraction of patient geometric size surrogates.

The errors from the ACR DIR approach will propagate through into the model‐based magnification correction and thus not provide a good estimate of the NDC compared to the gold standard as shown in Fig. 4. It is also possible that the gold standard estimates were inaccurate due to patient truncation in the CT axial image which correlates with the patient being too large for the AP CT localizer. Shown in Fig. 5(a) is an example of an outlier case where the patient has one or both arms down, and it is included into both the ACR DIR for CT localizers and the connected component analysis used to extract CT axial information. Figure 5(b) shows an example of a case where there is a device attached to the patient's chest area, and it would not be factored into the ACR DIR calculation, but would be factored into the CT axial patient size surrogate extraction method. A patient size surrogate that utilizes patient attenuation properties such as the water‐equivalent diameter (DW) would solve the issue in accounting for devices or other material place on the patient when comparing it to the gold standard. Table 3 shows the mean error of the effective diameter and SSDE from the ACR DIR and magnification corrected methods to the CT‐axial based approach along with the R^2^ values.

**Table 3:** The mean error of effective diameter and SSDE from the ACR DIR method with and without magnification correction to the CT axial‐based measurements.

Data set	Mean error (%) effective diameter	Mean error (%) SSDE	R^2^
ACR DIR	1.4	0.5	0.94
ACR DIR with magnification correction	4.4	5.3	0.78

The regression (R^2^) value is shown for both methods.

The limitation to our work is that we did not include a calculation of the water‐equivalent diameter (DW) for CT localizers. This can be done using the approach by Zhang et al., however, it would require a calibration with elliptical water phantoms using the same scanner.^18^ For the calibration method, the DW calculation requires an accurate measurement of the LAT dimension, therefore the model‐based magnification correction approach^9^ would provide an excellent estimate of these size surrogates and would yield an accurate DW. The data were acquired on a single scanner to demonstrate the magnification method. However since there is no dependence on absolute pixel values in the CT localizer, the model‐based magnification method will work for CT localizers on any vendor.

The model‐based method that accounts for geometric magnification reduces errors in size measurements. The normalized dose coefficients from the patient size surrogates calculated using the model‐based magnification approach are more accurate compared to the ACR DIR patient size surrogates extracted directly from the CT localizers. The thresholding based size methods fail for large and small patients and this renders inaccurate results for normalized dose coefficient measurements.

Key conclusions:

1. A novel magnification method can provide accurate estimates of geometric patient size surrogates which can be used to calculate SSDE prior to patient scanning.
2. The ACR DIR extraction method fails for bariatric (large) patients and smaller pediatric patients.

The model‐based magnification approach may be used on lateral and AP CT localizers to estimate patient dose, SSDE, prior to the CT scan.

## Conflicts of Interest

The author has no conflicts of interest to disclose.
